# Successful Laparotomy in the First Hour of Life

**Published:** 1897-06

**Authors:** 


### SUCCESSFUL LAPAROTOMY IN THE FIRST HOUR OF LIFE.

Professor Bayer recently performed a radical operation on a new-born infant with a very large umbilical hernia, rupture of the sac, and eventration of almost the entire large small intestines. He enlarged the mouth of the sac, replaced the intestines, excised the hernial sac, and closed the wound, using 20 grams of pure chloroform, which was well tolerated. Recovery was prompt and uneventful. The choice between Breuss' per-cutaneous ligature, Olshausen's extra-peritoneal method and the customary laparotomy, with excision of the sac and suture of the mouth, is in favor of the latter, which is the best and most reliable in cases like the above. There are thirty-three cases on record, with twenty-six recoveries; the present is the third on record performed during the first hour after birth, and the first in which there was rupture of the sac, which is an unconditional indication therefore for laparotomy in umbilical hernia.—*Prager Medical Woch.—Journal American Medical Association.*

